# Editorial: Women in thoracic surgery

**DOI:** 10.3389/fsurg.2022.1055021

**Published:** 2022-11-23

**Authors:** Paola Ciriaco

**Affiliations:** Department of Thoracic Surgery, IRCCS San Raffaele Scientific Institute, Vita-Salute San Raffaele University, Milan, Italy

**Keywords:** thoracic surgery, women, VATS, uniportal, training

**Editorial on the Research Topic** Women in thoracic surgery By Ciriaco P. (2022) Front. Surg. 9: 1055021. doi: 10.3389/fsurg.2022.1055021

Thoracic surgery, in its second century of existence, is experiencing an increase in the number of women and underrepresented minorities entering the field (1). Nowadays, women comprise nearly half of medical school graduates and, in parallel, the number of women in thoracic surgery has steadily increased (2, 3). Societies like Women in Thoracic Surgery and Women in General Thoracic Surgery, work to ameliorate the mentorship and education of women in this specialty across North America and Europe (4); still, numerous challenges persist for the successful career of women in thoracic surgery (5). Despite all the challenges, women in thoracic surgery are perfectly aligned with their male counterparts in terms of surgical and academic skills.

This Special Issue in Frontiers in Surgery brings together a series of high-quality scientific contributions that underline the versatility of the Authors concerning the pathologies addressed in the field of thoracic surgery.

Screening, timeless of diagnosis and treatment, represents an identification of the modern approach to lung cancer. Disparities based on ruralities have been analyzed in the paper by Minerva et al. Most studies suggest a delay in the diagnosis and treatment of lung cancer for rural residents. The Authors report a paradoxical result in their study, with a faster diagnosis in patients in rural areas, probably thanks to the active involvement of the general practitioner in their small country.

Thoracic surgery in the last twenty years is increasingly oriented toward minimally invasive surgery. As part of it, Video-Assisted Thoracic Surgery (VATS) offers patients, good results and great satisfaction (6). All thoracic surgery centers of a certain level, offer a training program for residents aimed at teaching new technologies for the treatment of old pathologies. The number of women in thoracic surgery has steadily increased, and they are more and more involved as the first surgeon in all kinds of operations.

Wang et al. presented in the Special Issue the treatment of a retrosternal goiter through a VATS subxiphoid modified approach. A retrosternal goiter is a relatively rare disease and there are many surgical approaches and strategies, such as a cervical neck incision, a combined thoracic incision, or a sternotomy. The use of VATS combined with a sternal retractor minimizes the patient's discomfort, and reduces postoperative complications, therefore reducing the hospital stay.

Women in thoracic surgery are also at the fore in following the improvement of existing mini-invasive technologies. VATS itself is changing such as turning into uniportal video-assisted thoracoscopy. Nachira et al. demonstrated the effectiveness of uniportal-VATS lobectomy compared to open surgery in early-stage lung cancer. In particular, nodal staging and surgical/oncological outcomes have been analyzed in the study, underlying the lower postoperative pain and shorter in-hospital stay compared to open surgery. Still, this is a challenging and demanding procedure to perform and the learning curve leads to an increase in the number of intraoperative complications (7).

Treatment of complications might be achieved through VATS too. Panza and colleagues describe the successful management of a chemothorax due to accidental drug injection into the pleural cavity for the wrong placement of an intravenous catheter.

VATS also allows for complex maneuvers, such as the removal of a displaced catheter, without the trauma of the open surgery. In addition, in this case, allowed the possibility to explore the chest cavity to exclude major trauma and a curative treatment employing lavage.

Thoracic surgery of modern times also pays particular attention to the Enhanced Recovery After Surgery (ERAS) (8). Nausea and vomiting affect a large part of patients undergoing thoracic surgery, slowing the postoperative recovery and increasing hospital stay. Jingli and colleagues contributed to the Special Issue by submitting a meta-analysis of a randomized controlled trial on the effect of dexmedetomidine on postoperative nausea and vomiting in patients undergoing thoracic surgery. They conclude that, regardless of the method of drug administration and the type of thoracic resection, dexmedetomidine, compared to a placebo, can reduce the onset of nausea and vomiting.

Although the literature demonstrates 360-degree female involvement in thoracic surgery, few women are in leadership positions (5). This might be attributed, in part, to the fact that women occupy more junior positions compared to men, but, in reality, indeed many of them do not achieve career progression. The lack of career progression may be due to different factors. A personal choice can occur in the cultural conviction that there is a family task that must be carried out by the woman and therefore irreconcilable with the type of work. More female surgeons reported not having a formal mentor and almost 70%, also reported never having been a mentor themselves (5). The presence of female mentors could help trainees to understand that there is a possibility to combine work and life. In several cases, a career progression is preferred for a man, and very often women have a lower salary compared to same-level men (2).

The national and international societies seem to show interest in addressing the gender disparity at a higher institutional level, and thoracic surgery is no anymore a male-dominated medical specialty (5). There are positive changes but much remains to be done starting with training. Statistics show that in 10 years more than half of surgeons will be women (2) and they will have more exposure and opportunities. Formal mentorship, salary disparities, and surgery work environment remain also important issues in achieving optimal results in terms of gender equity.
